# The Impact of Parent–Child Attachment on Self-Injury Behavior: Negative Emotion and Emotional Coping Style as Serial Mediators

**DOI:** 10.3389/fpsyg.2020.01477

**Published:** 2020-07-31

**Authors:** Yun Tao, Xiao-Yan Bi, Min Deng

**Affiliations:** ^1^College of Education Science and Management, Yunnan Normal University, Kunming, China; ^2^Key Laboratory of Educational Information for Nationalities, Kunming, China; ^3^Department of Psychoiogy, Honghe University, Mengzi, China; ^4^Faculty of Psychology, Southwest University, Beibei, China

**Keywords:** junior high school students, parent–child attachment, self-injury behavior, negative emotion, emotional coping style

## Abstract

In order to explore the relationship between parent–child attachment, negative emotion, emotional coping style, and self-injury behavior, 662 junior high school students in four junior middle schools in China’s Yunnan Province were investigated using a parent–child attachment questionnaire, adolescent negative emotion questionnaire, emotional coping style scale, and adolescent self-injury behavior scale. As a result, two mediate models were created to explain how parent–child attachment affects self-injury behavior. Negative emotion and emotional coping style play serial mediating roles in mother–child and father–child attachment models, respectively. The results show that negative emotion mediates between self-injury behavior and both father–child and mother–child attachment, while emotional coping style only functions between father–child attachment and self-injury behavior. By means of bootstrap analysis, negative emotion and emotional coping style have serial mediating roles concerning the impact of parent–child attachment on self-injury behavior. By comparison, the father–child and mother–child attachment have different mediating models: the former relies on emotional coping style, while the latter is associated with emotional experiences. This implies that parent–child attachment has different mechanisms in triggering self-injury behavior, which is in line with the hypothesis of attachment specificity.

## Introduction

Non-suicidal self-injury (hereafter referred to as NSSI or self-injury) is the direct, deliberate destruction of one’s own body tissue in the absence of suicidal intent, which is intended by the individual, rather than accidental (Klonsky and Olino, 2008; Nock and Favazza, 2009). The incidence of self-injury among the general population is 1 ≤ 4% (Sleuwaegen et al., 2017); however, adolescents with a history of self-injury are up by 30–40% (Yulong et al., 2016; Cerutti et al., 2018). Domestic studies show that the proportion of self-injury behavior among teenagers is over 20% (Feng, 2008; Ying et al., 2013; Yinqgian et al., 2015). Thus, there is clearly a high occurrence of self-injury behavior, especially among adolescents.

Self-injury behavior is a negative way for individuals to deal with current problems (Sornberger et al., 2013), and causes great harm to adolescents’ mental health (Guvendeger et al., 2017; Baetens et al., 2019). At the same time, self-injury behavior is also a risk factor leading to suicide. In the absence of intervention, it may lead to a high possibility of suicide among people (Guan et al., 2012; Ge et al., 2014). The Biosocial Model also holds that an ineffective family environment is an important cause of self-injury behavior, and parents’ neglect of children’s emotion and abuse can most directly cause self-injury behavior (Thomassin et al., 2016). The parent–child relationship is unavoidably related to the formation and sustainable development of self-injury behavior in junior high school students. In the face of increasingly severe self-injury behavior, previous research has mainly investigated the effects of adverse family environments on adolescents’ self-injury behavior, such as early traumatic experiences, emotional susceptibility, personality characteristics, stressful life events, family conflicts, etc. (Fuxia et al., 2018; Buser et al., 2019). A strong parent–child attachment with an emotional link in their relationship may to some extent prevent junior high school students from self-injury behavior. However, the mechanism of how parent–child attachment affects adolescents’ self-injury behavior still remains unclear. Therefore, this paper mainly discusses the impact of parent–child attachment on one’s self-injury behavior as well as the mechanism behind it, in an attempt to offer targeted suggestions for the prevention and intervention of self-injury behavior in junior high school students.

Parent–child attachment is a relatively stable relationship model formed in the communication between children and their parents, which can significantly influence children’s social communication and mental health during their growth (Yulong, 2015). A study demonstrates that there are various and unique associations between family based risk factors and self-injury behavior, as well as addictive features (Martin et al., 2016). It is worth noting that the experience of adverse family life is correlated with self-injury behavior. In addition, another study shows that high-quality family relationships, such as parent–child trust and parent–child communication, were linked to fewer discipline violations, less antisocial behavior (Burk and Laursen, 2010), and lower levels of anxiety, depression (Nadorff et al., 2014), and loneliness, which may further contribute to middle school students’ higher emotional levels (Al-Yagon, 2012), their seeking help, and more positive ways to adapt to society, thus reducing the probability of self-injury. On the contrary, the higher the degree of parent–child alienation, the higher the risk of self-injury (Weihua, 2016). In the period of adolescence, junior high school students are going through drastic changes both psychologically and physiologically, being faced with a series of adaptation problems, including physiological maturity, cognitive development, and social role transformation (Guoliang and Yu, 2018). Thus, when they fail to cope with family relationships, they may alleviate their pain by means of self-injury behavior (Victor et al., 2012). Fuxia et al., 2018 has pointed out that parent–child attachment is an important factor in adolescents’ self-injury behavior (Fuxia et al., 2018). In addition, recent studies have demonstrated that poor-quality parent–child attachment can increase the incidence of self-injury behavior (Honglei et al., 2018). Interpersonal or systematic models have also pointed out that self-injury is the result of family dysfunction, and that the family environment of certain individuals will unconsciously support or strengthen their self-injury behavior (Crouch and Wright, 2004). Moreover, studies find that fathers and mothers play different roles in parent–child communication; the father spends relatively less time interacting with their children, and tends to engage in more physical and outdoor activities, while mothers invest time and are involved in more caring and household interactions (Oliveri et al., 2018). Consequently, it is possible that father–child and mother–child relationships represent different variables that affect the self-injury behavior of junior high school students. Previous research says that children have fewer problematic behaviors with a healthy father–child relationship (Hardcastle et al., 2018). Meanwhile, the attachment specific hypothesis further indicates that when children undergo attachment experience with different caregivers, the multiple layers of attachment formed boast a non-differentiated degree of influence on children’s social and psychological development (Zborowski and McNamara, 1998); i.e., the influence of father–child and mother–child attachment on children’s development is not superimposed or comprehensive, but independent from one another. Do father–child and mother–child attachments have the same impact on self-injury behavior? We will explore their respective mechanisms.

At present, the mechanism of how parent–child attachment influences self-injury behavior stills remains unclear and requires further exploration. Two main theories used to explain self-injury behavior are the interpersonal or systematic models, as well as the emotion regulation model (Yulong et al., 2016). Interpersonal or systematic models emphasize the impact of the family environment on self-injury behavior, demonstrating the influence of the family system on adolescent problematic behavior, especially from the perspective of parent–child relationships (Crouch and Wright, 2004). By contrast, the emotion regulation model shows that individual self-injury behavior is a behavioral strategy to deal with negative emotions or reduce pain from the angle of emotional management (Messer and Fremouw, 2008). However, these two models do not explore the negative emotions caused by parent–child relationship in the family system, so they fail to fully reveal the effects of parent–child attachment on negative emotions, emotional coping style, and self-injury behavior. In other words, the question is, as negative emotions are generated by poor parent–child relationship, why are children unable to manage their emotions effectively, and why is parent–child attachment not protective to self-injury behavior? A possible explanation is that, due to negative emotions, individuals choose self-injury behavior as a coping method, which is influenced by the parent–child relationship. A poor attachment relationship may strengthen the connection between negative emotions and self-injury behavior; good attachment weakens it. Adolescents, lacking in emotional regulation and control, can possibly resort to self-punishment when facing environment changes and negative emotions. This kind of emotional coping style gives junior high school students the tendency to adopt self-injury behavior instead of other approaches when faced with poor family relationships (Glassman et al., 2007). Therefore, in order to examine how and when father–child and mother–child attachment were linked to adolescents’ self-injury behavior, negative emotion and emotional coping style are listed as possible mediating factors.

Negative emotion is a general term for subjective stress and unpleasant experiences (Watson et al., 1988), which reflects individual differences in emotional stability (Watson, 2000). Previous studies have found that high-quality parent–child attachments can reduce the influence of negative emotions on individuals, and vice versa (Yinqgian et al., 2015). Yan et al. (2018) have also discovered that good parent–child relationships can meet individual’s emotional needs, while low-quality relationships may contribute to negative emotions as the result of parent–child alienation (Yan et al., 2018). Furthermore, some studies have suggested that mother–child attachment can be directly employed to predict negative emotions, while some other studies have showed that the father–child attachment has no such obvious effect, which means the impact of the former outweighs that of the latter (Yinqgian et al., 2015). Thus, all these studies have demonstrated that unhealthy father–child and mother–child attachment can directly trigger junior high school students’ negative emotions, and that the influencing mechanisms are actually different. On the other hand, negative emotions may bring about self-injury behavior. A study has found that individuals with high negative emotions can probably relieve their emotional burdens through self-injury behavior, and may weaken the psycho-social function of junior high school students, leading to a series of problems inducing depression and anxiety (Yulong et al., 2019). The experiential avoidance model and emotional management model on self-injury behavior also proposes that individuals will eliminate their unhealthy emotions through self-injury behavior when lacking an effective emotional management ability or to avoid unpleasant emotions (Paivio and Mcculloch, 2004; Chapman et al., 2006); i.e., individuals manage their negative emotions by means of self-injury behavior (Quanquan et al., 2017). Further, more studies have also confirmed that there is a significant positive correlation between negative emotion and self-injury behavior (Yulong et al., 2019). Generally speaking, negative emotion, as a mediate variable, has a wide range of impacts on individual’s cognition, motivation, and social behavior. Therefore, based on the model above, it is assumed that negative emotion is to some extent related to the self-injury behavior of junior high school students. Thus, we expected negative emotion may play a mediating role between father–child and mother–child attachment and self-injury behavior.

Coping style refers to the strategies adopted by individuals to relieve stress under pressure (Runxin and Jianmei, 2016), i.e., problem and emotional coping styles (Folkman and Lazarus, 1980). Some researchers have pointed out that people are more likely to adopt emotional coping methods such as enduring, escaping, venting, fantasy, and denial, when people believe there is no way out (Folkman and Lazarus, 1980). Probing into literature, Guerreiro et al. have found that self-injury behavior is linked to emotional-directed coping strategies (Guerreiro et al., 2013). People with self-injury behaviors tend to take emotional responses, and individuals constantly resorting to emotional responses are more likely to injure themselves when dealing with adverse emotions (Cramer et al., 2017). With an observation of 965 adolescents, Castro and Kirchner (2017) have made it clear that adolescents with multiple self-injury behaviors would employ more coping strategies like venting, which has therefore proven a risk factor for self-injury. The evidence above shows that emotional coping style is closely associated with self-injury behavior. In a family, an adverse parent–child relationship is the direct cause of emotional coping styles (Qiangqin, 2010). As junior high school students lack social experience, when they suffer an adverse parent–child relationship, they most probably have a sense of inability, not knowing how to change their situation and being apt to resort to emotional coping methods. Attachment theory has also proposed that the secure attachment formed earlier between children and their caregivers can affect the coping styles of junior middle school students (Davila and Levy, 2006). Meanwhile, Qiangqin, 2010 has also found that father–child attachment and mother–child attachment could both significantly negatively predict emotional coping style (Qiangqin, 2010). Considering this, we expected emotional coping style may play a mediating role between father–child and mother–child attachment and self-injury behavior.

Moreover, there is also a close positive correlation between negative emotion and emotional coping style. Domestic researchers have discovered that emotional problems are positively related to their coping styles; foreign studies suggest that negative emotion can lead to individuals reacting with such coping styles as avoiding, denying, and enduring, where emotional coping style is considered as a “response to emotion” (Folkman and Lazarus, 1980). To sum up, it is believed that negative emotion may affect emotional coping style; therefore, it is essential to examine the serial mediating roles of these two factors in the correlations between father or mother–child attachment and self-injury behavior.

The present study’s main objective is to analyze the different roles of father–child and mother–child attachment in the development of self-injury behavior of adolescents. Thus, we aim to observe the associations between parent–child attachment, negative emotion, emotional coping style, and self-injury behavior. Lastly, we will analyze the pathways through which father–child and mother–child attachment is linked to self-injury behavior. Therefore, this research has been guided by four objectives. First, we expected parent–child attachment to be associated with self-injury behavior, and there to be a difference between the effects of father–child attachment and mother–child attachment on self-injury behavior. Second, we anticipated that negative emotion may play a mediating role between father–child and mother–child attachment and self-injury behavior. Third, we expected emotional coping style may play a mediating role between father–child and mother–child attachment and self-injury behavior. Finally, we expected there to be a serial mediating role played by negative emotion and emotional coping style in the correlations between father or mother–child attachment and self-injury behavior.

## Materials and Methods

### Subjects and Procedure

Participants for this study were recruited from the junior high schools in China’s Yunnan Province. Six hundred and seventy students were approached to take part in this study. The final sample consisted of 309 (46.7%) boys and 353 (53.3%) girls. In reference to the participants’ schooling, 198 were in the first year of junior high school, 204 in the second year, and 260 in the third. The age range was between 13 and 18 (*M* = 15.04, SD = 1.92).

The data were collected by means of cluster random sampling in the four junior high schools. Ethical approval for this project was given by the executive council of Yunnan Normal University. The adolescents signed their informed consent. Data collection occurred in a classroom context, in the presence of the supervising researcher who, in a succinct manner, administered the standard instructions, including the general objectives of the study as well as guaranteeing free will, privacy, anonymity, and confidentiality of all the information provided.

### Measures

#### Parent–Child Attachment Questionnaire (Inventory of Parent and Peer Attachment, IPPA)

The parent–child attachment questionnaire, compiled by Armsden and Greenberg (1987), was translated by Wendao et al. (2008) for use in China. This questionnaire in its revised form is composed of 75 questions that are distributed in three sub-questionnaires, from which we adopt two sub-questionnaires in this study: the father–child attachment questionnaire—“*I think my father is a competent father*”; the mother–child attachment questionnaire—“*My mother respects my feelings”*. Each father–child and mother–child attachment questionnaire included 25 items, which were divided into three dimensions: trust, communication, and alienation. For each item, there are five answer options given for the father and the mother separately, in which the responses are presented on a *Likert* style scale that varies between 1 (Totally Disagree) and 5 (Totally Agree). In this study, the *Cronbach’s alpha* coefficients of each dimension on the father scale were 0.89, 0.87, and 0.74, respectively, and those on the mother scale were 0.88, 0.87, and 0.72 separately.

#### Adolescent Negative Emotion Questionnaire (Negative Affect Scale, PNAS)

The negative emotion questionnaire translated into Chinese by Wenfeng and Jianxin (2004) from the original version by Bradburne and Tyrrell (1969) was used with the objective of evaluating the negative emotional experience from the last 6 months. We used the sub-questionnaire of the negative affect scale. It includes six items—“*I feel like I’m upset for some reason.”* For each item, there are four answer options, presented on a *Likert* style scale, which varies between 1 (Never) and 4 (Always). The higher the score, the more intense the negative emotion. The psychometric studies performed in the present sample revealed a *Cronbach’s alpha* coefficient of 0.79.

#### Emotional Coping Style Scale (Coping Style Scale, CSS)

The coping style scale by Shulin et al. (2000) was used to evaluate adolescents’ emotional response to problems or troubles. This questionnaire in its revised form consists of 36 questions that are distributed in two sub-questionnaires. We used the sub-questionnaire of the emotional coping style scale (17 items), which presents itself in four dimensions: endurance (4 item)—“*I buried the unpleasant things in my heart*,” escape (4 item)—“*Refuse to believe that bad things have happened*,” venting emotion (4item)—“*You will be very upset and vent to your family and friends when you can’t solve the problem*,” and fantasy denial (5item)—“*Love to do something unrealistic to eliminate trouble*.” For each item, there are four answer options, presented on a *Likert* style scale, which vary between 1 (Not Adopt) and 4 (Always Adopt). The psychometric studies performed in the present sample revealed a *Cronbach’s alpha* coefficient of 0.81.

#### Adolescent Self-Injury Behavior Scale

The adolescent self-injury behavior scale, translated into Chinese by Fang et al. (2015) from the original version of the Ottawa self-injury scale, was used with the objective of evaluating the adolescents’ self-injury behavior from the past year. It includes 10 items—“*Deliberately burying your head in the water*.*”* The frequency of self-injury behavior in each way was set to 0 times, 1 time, 2–4 times, or 5 times or more, and the total score of the items was calculated as a continuous variable. The psychometric studies performed in the present sample revealed a *Cronbach’s alpha* coefficient of 0.95.

#### Analysis Method

The present study is of a cross-sectional nature given that the set of measurements were all carried out at the same moment in time. Data treatment was carried out using the statistical program SPSS—Statistical Package for Social Sciences, version 22.0 for Windows, and the mediate model was tested with AMOS 21.0. In order to test the mediating effect, the percentile Bootstrap method for deviation correction was used, which is recommended by Jie and Minqiang (2012).

## Results

### Common Method Deviation Test

In order to control the common method deviation, we told all participants that the questionnaires were filled out anonymously, and all the data were strictly confidential and only used for scientific research. The Harman single factor method was adopted to test common method deviation, and thus the exploratory factor analysis for all variables referred to in the research was carried out. The results show that the eigenvalues of 12 factors are greater than 1, and the variation explained by the first factor is 15.76%, less than 40%, which means that there is no common method deviation in this study (Hao and Lirong, 2004).

### Description Statistics and Correlation Analysis

A total of 662 participants were recruited for the study, with the response rate being 98.8%.

Among the respondents, 64.3% of males and 65.1% of females never committed self-injury behavior; 58.5 and 70.9% of those who never self-injured were from rural and urban areas respectively; 79.4, 71.4, and 78.6% of the never-self-injured students were in their first, second, and third year, respectively (Table 1).

**TABLE 1:** Frequency distribution of NSSI.

**Variables**	**NSSI**	
	**Never (%)**	**Occasional to frequent (%)**
Gender		
Female	64.3	35.7
Male	65.1	34.9
Residence		
Rural	58.5	41.5
Urban	70.9	29.1
Year of Study		
First	79.4	20.6
Second	71.4	28.6
Third	78.6	21.4

The results of the inter-scale correlations and the respective averages and standard deviations, reported in Table 2, allow us to state that there are correlations between the variables being studied. The correlation analysis showed that there was positive correlation between mother–child and father–child attachment, but negative correlation with negative emotion, emotional coping style, and self-injury behavior. Meanwhile, negative emotion, emotional coping style, and self-injury behavior were positively correlated with each other.

**TABLE 2:** Averages, standard deviations, and correlation coefficients of variables.

**Variables**	***M***	**SD**	**1**	**2**	**3**	**4**
1. Mother–child attachment	2.96	0.48				
2. Father–child attachment	2.85	0.47	0.74**			
3. Negative emotion	2.37	0.71	−0.51**	−0.34**		
4. Emotional coping style	2.31	0.48	−0.35**	−0.47**	0.50**	
5. Self-injury behavior	0.89	0.86	−0.32**	−0.30**	0.39**	0.41**

*p < 0.05, **p < 0.01, ***p < 0.001, the same as below.

### Model Verification and Analysis

The mediating effects of the quality of negative emotion and the emotional coping style variable were calculated using the Structural Equations Model, through the realization of the steps of the percentile Bootstrap method. The analysis of the mediating roles of the negative emotion and emotional coping style were carried out, taking into account all of the following dimensions: the negative emotion, the emotional coping style (endurance, escape, venting emotion, fantasy, and denial) as serial mediators of both the parent–child attachment (trust, communication, and alienation), and self-injury behavior.

In regard to the model test that verified the parent–child attachment and self-injury behavior, the model test verified that initially. The results are listed as below. The father–child attachment model fits well (χ*^2^/df* = 4.21, RMSEA = 0.06, NFI = 0.98, IFI = 0.97, CFI = 0.99, SRMR = 0.04), and the attachment can significantly negatively affect self-injury behavior (β = −0.40, *p* < 0.001). The mother–child attachment model is also well fitted (χ^2^*/df* = 4.09, RMSEA = 0.07, NFI = 0.99, IFI = 0.98, CFI = 0.97, SRMR = 0.04), and this attachment can significantly negatively affect self-injury behavior (β = −0.30, *p* < 0.001). In summary, the father–child attachment and mother–child attachment can both significantly negatively affect self-injury behavior.

#### Verification and Analysis of Father–Child Attachment Mediate Model

**FIGURE 1:**
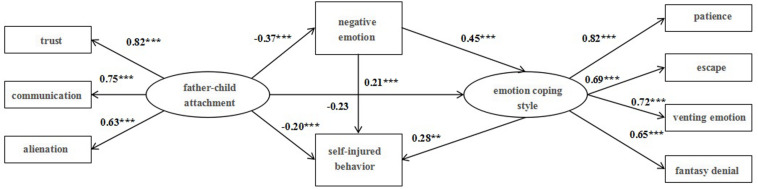
Father–child attachment mediate model.

The fitting degrees of the structural model and the data of father–child attachment were tested, and the results show that the fitting indexes of the model are sound: χ*^2^/df* = 2.07, RMSEA = 0.04, NFI = 0.95, IFI = 0.97, CFI = 0.96, SRMR = 0.03, as displayed in Figure 1. The normalized load of all the observed variables on corresponding latent variables is between 0.51 and 0.73, and the composite reliability values are all larger than the standard value of 0.6, indicating that the measurement model has reached the ideal standard, and the observed variables can well reflect the corresponding latent variables. Thus, the structural model can be further tested. With negative emotion and emotion coping style taken into consideration, the significant paths are as follows: father–child attachment has a direct negative effect on negative emotion (β = −0.37, *p* < 0.001) and self-injury behavior (β = −0.20, *p* < 0.001); negative emotion positively affects emotional coping style (β = 0.45, *p* < 0.001) and self-injury behavior (β = 0.21, *p* < 0.001); and emotional coping style positively affects self-injury behavior (β = 0.28, *p* < 0.01).

Furthermore, the percentile Bootstrap method of deviation correction was employed to test mediating effects (Jie and Minqiang, 2012). 5000 Bootstrap samples were extracted from the original data (*N* ≤ 662) by repeated random sampling, and the Bootstrap 95% confidence interval of the mediating effect was calculated. The results, as are demonstrated in Table 3, show that the Bootstrap 95% confidence interval of the mediating paths above do not include 0, which means that father–child attachment can affect self-injury behavior through the mediating role of negative emotion and emotional coping style, and that negative emotion and emotional coping style play a serial mediating role between father–child attachment and self-injury behavior.

**TABLE 3:** Bootstrap analysis to significance test of mediate effect.

**Mediate path**	**Indirect effect quantity**	**Lower limit of Boot CI**	**Upper limit of Boot CI**
Father–child attachment→negative emotion→self-injury behavior	−0.19***	–0.13	–0.06
Father–child attachment→emotional coping style→self-injury behavior	−0.08**	–0.11	–0.10
Father–child attachment→negative emotion→emotional coping style→self-injury behavior	−0.09***	–0.08	–0.04

The lower limit and upper limit of Boot CI respectively refer to the lower and upper limits of the 95% confidence interval of the indirect effect estimated with percentile Bootstrap method to correct deviation; all values retain two decimal places, the same as below. *p < 0.05, **p < 0.01, ***p < 0.001.

#### Verification and Analysis of Mother–Child Attachment Mediate Model

**FIGURE 2:**
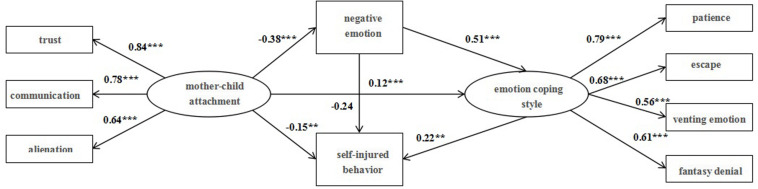
Mother–child attachment mediate model.

The fitting degrees of the structural model and data of mother–child attachment were tested, and the results indicate that the fitting indexes are sound: χ^2^/*df* = 3.08, RMSEA = 0.06, NFI = 0.91, IFI = 0.94, CFI = 0.95, SRMR = 0.04, as represented in Figure 2. The observed variables can well reflect the corresponding latent variables (the calculation method is the same as above); therefore, the structural model can be further tested. With negative emotion and emotion coping style taken into account, the significant paths are as follows: mother–child attachment can directly negatively affect negative emotion (β = −0.38, *p* < 0.001) and self-injury behavior (β = −0.15, *p* < 0.001); negative emotion can positively affect emotional coping style (β = 0.51, *p* < 0.001) and self-injury behavior (β = 0.12, *p* < 0.001); and emotional coping style can positively affect self-injury behavior (β = 0.22, *p* < 0.01).

In addition, the percentile Bootstrap method of deviation correction is adopted to test mediating effects. The results, as are listed in Table 4, demonstrate that the 95% confidence interval of the mediating paths above do not include 0, indicating that the mediating effect is significant. In other words, mother–child attachment could affect self-injury behavior through the mediating effect of negative emotion, with negative emotion and emotional coping style playing serial mediating role between mother–child attachment and self-injury behavior.

**TABLE 4:** Bootstrap analysis to significance test of mediate effect.

**Mediate path**	**Indirect effect quantity**	**Lower limit of Boot CI**	**Upper limit of Boot CI**
Mother–child attachment→negative emotion→self-injury behavior	−0.10***	–0.13	–0.06
Mother–child attachment→negative emotion→emotional coping style→self-injury behavior	−0.09***	–0.21	–0.05

*p < 0.05, **p < 0.01, ***p < 0.001.

## Discussion

### The Direct Effect of Parent–Child Attachment on Self-Injury Behavior

This study finds that father–child and mother–child attachment of junior high school students can both directly negatively influence self-injury behavior, which conforms to the basic view of interpersonal or systematic models (Crouch and Wright, 2004). This also supports the conclusion of previous studies; i.e., adverse parent–child attachment will lead to the students’ self-injury behavior (Martin et al., 2016; Gromatsky et al., 2017). Family relationships are critical to adolescents, and they tend to adopt self-injury instead of other means to ease the tension and stress once the relationships fall ill. Some studies have discovered that a high-quality parent–child relationship is a protective factor. When there is a poor quality parent–child relationship, children are more likely to resort to self-injury (Glenn and Klonsky, 2010), which means that a healthy parent–child relationship is an essential factor to prevent junior high school students from self-injury behavior. In addition, the higher the score of parent–child attachment, the lower the score of individual self-injury behavior, i.e., the better the quality of attachment, the more trust and cooperation between parents and children, and the lower the possibility of individual self-injury behavior.

### The Mediating Role of Negative Emotion and Emotional Coping Style

This research finds that negative emotion can mediate between father–child and mother–child attachment and self-injury behavior, i.e., parent–child attachment can not only directly, but also indirectly, effect self-injury behavior through negative emotion. It also testifies the main principles of theexperience avoidance model and emotion management model (Paivio and Mcculloch, 2004; Chapman et al., 2006). It demonstrates that parent–child relationships can induce negative emotion, which will further trigger the occurrence of self-injury, both being consistent with the previous studies (Klonsky et al., 2003; Feng, 2008). When the individuals lack the ability to manage their negative emotion effectively, they try to relieve the emotion through self-injury. Thus, it is certain that negative emotion increases the risk of self-injury behavior.

Additionally, father–child attachment can be negatively associated with emotional coping style, and the latter further effects self-injury behavior, which is in line with the results of previous studies (Qiangqin, 2010; Castro and Kirchner, 2017). Owing to the lack of experience to cope with social problems, junior high school students do not know how to deal with challenging problems and tend to adopt emotional coping styles after suffering from father–child conflict. However, the emotional coping style puts more emphasis on emotion itself, which is not conducive to solving the problems, but reinforces the negative emotion and even leads to self-injury. Studies have shown that the students being accustomed to the emotional coping style are slow at communication, with a significantly higher level of anxiety and depression compared with the general population, and previous studies have also proven that the continuous negative emotional coping style can result in self-injury (Konick and Gutierrez, 2005). It shows that a poor quality father–child relationship will increase the chance of emotional coping style by junior high school students, thus increasing their self-injury behavior. In terms of mother–child attachment, however, a poor relationship cannot directly affect emotional coping style, which is inconsistent with former studies (Qiangqin, 2010). This is because mothers, as the main supporter of the children’s lives, have established a close relationship with their children from an early age. Such a bond tends to be more emotionally connected. Therefore, the unhealthy mother–child attachment does not easily lead to an emotional response.

At the same time, negative emotion and emotional coping style play serial mediating roles between father–child and mother–child attachment and self-injury behavior. It is believed that junior high school students can have negative emotions due to poor quality parent–child attachment (Yulong et al., 2016), under the influence of which individuals resort to a series of solutions to adapt to their negative emotions. With a sense of inability in emotional processing, they naturally surrender to the emotional coping style, which further contributes to self-injury behavior (Folkman and Lazarus, 1980; Castro and Kirchner, 2017).

### The Mediating Model of Father–Child and Mother–Child Attachment

As children grow up, fathers will use games and set challenges to encourage children to face dangers, overcome difficulties, and promote children’s social adaptability (Paquette, 2004), which may serve as an effective indicator of adolescents’ psychological elasticity (Xiaoyan et al., 2011); mothers, however, tend to be more emotionally connected with their children. Recent research has also suggested that father–child attachment and mother–child attachment have different roles in the parent–child relationship. Father–child attachment has a more significant role in promoting children to cope with challenges and emotions, while mother–child attachment is mainly reflected in emotional experience. Therefore, the intermediate model in this study is in line with the attachment specific hypothesis.

The mother–child attachment model tends to affect emotional experience, while the father–child attachment model would affect behavioral coping style. This shows that a child’s emotional connection with his mother is the basis and starting point for exploring the outside world and will affect his whole life. This is because in the early attachment relationship, infants and children internalize the interaction experience with their attachment objects into their internal working mode. When they contact others later, the internal working model will guide his behavior performance, which also supports the attachment theory. Fathers are often a role model who encourages their children to face and solve problems, which is line with previous explanations (Gottman, 1998). The reason for this difference may come from traditional family rearing pattern of “strict father and gentle mother,” and adolescents generally hold the stereotype that mothers are more sensitive, gentle, and modest, while fathers are decisive, independent, and vigorous, so the latter is usually imitated by children (Chuanhua et al., 2003). This study proves that father–child attachment is highly predictive of individuals’ stress response, which means that father–child attachment, via words and deeds, may change the way junior high school students cope with their emotions. Maybe fathers with bad behaviors such as alcoholism, smoking, etc., are more likely to pass their emotional coping styles to their children; however, mother–child attachment does not have this effect, i.e., mothers emotional coping style has less of an effect on children. In other words, mother–child attachment more depends on emotional communication and expression, so an unhealthy mother–child relationship brings more negative emotional feelings. In a word, the quality of mother–child attachment and father–child attachment embodies the trust and cooperation between parents and children, but they have different connotations (Yan, 2016). For example, parent–child attachment mainly promotes the emotional communication between parents and children through caring and nurturing behaviors, while parent–child attachment mainly reflects the quality of parent–child attachment by setting an example and giving confidence. The connotation of this attachment is also reflected in the process of emotional adaptation and emotional management. Mothers show more emotional warmth and understanding, but fathers demonstrate more problem-solving, so mother–child attachment establishes relationship quality through positive emotions, while father–child attachment reflects relationship quality through problem-solving and positive response.

Parent–child attachment plays an important positive role in the healthy development of individuals; however, unhealthy attachment can destroy the ability for emotional adaptation and regulation. It not only directly affects the self-injury behavior of individuals, but also influences the self-injury behavior through negative emotions and negative emotional coping styles. This study provides a new angle for the intervention and education of self-injury behavior, i.e., from the perspective of attachment theory, paying attention to the impacts of father–child attachment and mother–child attachment work models on individual self-injury behavior. Attachment mode not only has gender differences, but also differences between safe attachment and non-safe attachment. Future research should further explore the relationship between non-safe attachment individuals and the relationship among negative emotion, emotional coping style, and self-injury behavior. In addition, there are some shortcomings in the study as well. First, as a cross-sectional study, the relationship between different attachment types and junior high school students’ self-injury behavior could not be examined in more detail. Future research may use tracking design to deepen this issue. Second, this study has only examined the impact of parent–child and mother–child attachment on self-injury behavior, without referring to peer attachment. In fact, junior high school students’ attachment targets are not limited to parents; peer attachment is also of huge importance. Studies have suggested that the role of peer attachment has even surpassed that of parent–child attachment in their development (Bogaerts et al., 2006), but there are other different perspectives, so future research may further explore the impact of peer attachment on junior high school students’ self-injury behavior. Third, the measurement of each variable is entirely based on the self-report of junior high school students; the method of measuring self-injury behavior specifically is relatively single, and there may be social approval effects. Thus, future research may consider combining multiple methods to obtain more comprehensive data. Fourth, the measured groups are all students, and the sample size is relatively small. In later research, more group samples could be considered for investigation.

## Conclusion

First, mother–child and father–child attachment directly negatively influences self-injury behavior of junior high school students. Second, father–child attachment affects self-injury behavior through separate mediation of negative emotion, through separate mediating effects of emotional coping style, and r through serial mediating effects of negative emotion and emotional coping style. Third, mother–child attachment affects self-injury behavior through separate mediation of negative emotion, and through serial mediating effects of negative emotion and emotional coping style.

## Data Availability Statement

The datasets generated for this study are available on request to the corresponding author.

## Ethics Statement

Ethical review and approval was not required for the study on human participants in accordance with the local legislation and institutional requirements. Written informed consent to participate in this study was provided by the participants’ legal guardian/next of kin. Written informed consent was obtained from the individual(s), and minor(s)’ legal guardian/next of kin, for the publication of any potentially identifiable images or data included in this article.

## Author Contributions

YT conceived the study, participated in its design, carried out the study, performed the statistical analysis, and drafted the manuscript. MD and X-YB supervised the study, participated in the design of the study, helped to draft the manuscript, and revised the manuscript critically. All authors read and approved the final manuscript.

## Conflict of Interest

The authors declare that the research was conducted in the absence of any commercial or financial relationships that could be construed as a potential conflict of interest.

## References

[B1] Al-Yagon M. (2012). Adolescents with learning disabilities: socioemotional and behavioral functioning and attachment relationships with fathers, mothers, and teachers. *J. Youth Adolesc.* 41 1294–1311. 10.1007/s10964-012-9767-6 22528372

[B2] Armsden G. C. Greenberg M. T. (1987). The inventory of parent and peer attachment: individual differences and their relationship to psychological well-being in adolescence. *J. Youth Adolesc.* 16 427–454. 10.1007/bf02202939 24277469

[B3] Baetens I. Claes L. Onghena P. (2019). Non-suicidal self-injury in adolescence: longitudinal associations with psychological distress and rumination. *J. Abnorm. Child Psychol.* 47 1569–1581. 10.1007/s10802-019-00531-8 30900112

[B4] Bogaerts S. Vanheule S. Leeuw F. Desmet M. (2006). Recalled parental bonding and personality disorders in a sample of exhibitionists: a comparative study. *J. Forensic Psychiatry Psychol.* 17 636–646. 10.1080/14789940600915701

[B5] Bradburne A. F. Tyrrell D. A. J. (1969). The propagation of “coronaviruses” in tissue-culture. *Arch. Gesamte. Virusforsch.* 28 133–150. 10.1007/BF01249379 4984061 PMC7086760

[B6] Burk W. J. Laursen B. (2010). Mother and adolescent reports of associations between child behavior problems and mother-child relationship qualities: separating shared variance from individual variance. *J. Abnorm. Child Psychol.* 38 657–667. 10.1007/s10802-010-9396-z 20217213 PMC2880240

[B7] Buser T. J. Pertuit T. L. Muller D. L. (2019). Non-suicidal self-injury, stress, and self-differentiation. *Adultspan J.* 18 4–16.

[B8] Castro K. Kirchner T. (2017). Coping and psychopathological profile in nonsuicidal self-injurious chilean adolescents. *J. Clin. Psychol.* 74 10.1002/jclp.22493 28586528

[B9] Cerutti R. Zuffianò A. Spensieri V. (2018). The role of difficulty in identifying and describing feelings in non-suicidal self-injury behavior (NSSI): associations with perceived attachment quality, stressful life events, and suicidal ideation. *Front. Psychol.* 9:318. 10.3389/fpsyg.2018.00318 29593617 PMC5859383

[B10] Chapman A. L. Gratz K. L. Brown M. Z. (2006). Solving the puzzle of deliberate self-harm: the experiential avoidance model. *Behav. Res. Ther.* 44 0–394. 10.1016/j.brat.2005.03.005 16446150

[B11] Chuanhua G. Huichang C. Jingjing X. (2003). The early family environment and parental rearing style of creative figures in modern Chinese society. *Psychol. Dev. Educ.* 12 17–22.

[B12] Cramer R. J. La Guardia A. C. Bryson C. Morgan K. (2017). The intersection of non-suicidal self-injury and suicide-related behavior: patterns of elevated risk and implications for college mental health. *J. Am. Coll. Health* 65 363–371. 10.1080/07448481.2017.1312416 28362249

[B13] Crouch W. Wright J. (2004). Deliberate self-harm at an adolescent unit:A qualitative investigation. *Clin. Child Psychol. Psychiatry* 9 185–204. 10.1177/1359104504041918

[B14] Davila J. Levy K. N. (2006). Introduction to the special section on attachment theory and psychotherapy. *J. Consult. Clin. Psychol.* 74 989–993. 10.1037/0022-006x.74.6.989 17154729

[B15] Fang Z. Wenhong C. Zeping X. (2015). A study on the reliability and validity of Ottawa self-injury questionnaire in Chinese. *J. Shanghai Jiaotong Univ.* 35 460–464.

[B16] Feng Y. (2008). *The Relationship Between Self-Injury Behavior and Individual Emotional Factors and Family Environment Factors in Adolescents.* Doctoral dissertation, Central China normal University, Wuhan.

[B17] Folkman S. Lazarus R. S. (1980). An analysis of coping in a middle-aged community sample. *J. Health Soc. Behav.* 21:219 10.2307/2136617 7410799

[B18] Fuxia R. Lixia Y. Shujian F. Liming Z. Hanmei T. Yanyan F. (2018). Parent-child attachment of middle school students and its relationship with non-suicidal self-injury behavior. *Sch. Health China* 293 47–53.

[B19] Ge Y. Yixin X. Jing Z. Weihong W. (2014). Analysis of self-injury behavior and related factors of college students in Chongqing. *Sch. Health China* 35 1679–1681.

[B20] Glassman L. H. Weierich M. R. Hooley J. M. Deliberto T. L. Nock M. K. (2007). Child maltreatment, non-suicidal selfinjury, and the mediating role of self-criticism. *Behav. Res. Ther.* 45 2483–2490. 10.1016/j.brat.2007.04.002 17531192 PMC2034449

[B21] Glenn C. R. Klonsky E. D. (2010). The role of seeing blood in non-suicidal self-injury. *J. Clin. Psychol.* 66 466–473. 20140887 10.1002/jclp.20661

[B22] Gottman J. M. (1998). “Toward a process model of men in marriages and families,”in *Men in Families: When Do They Get Involved? What Difference Does it Make?*, Booth A. Crouter A. C. (New Jersey: Lawrence Erlbaum Associates Publishers).

[B23] Gromatsky M. A. Waszczuk M. A. Perlman G. Salis K. L. Klein D. N. Kotov R. (2017). The role of parental psychopathology and personality in adolescent non-suicidal self-injury. *J. Psychiatr. Res.* 85 15–23. 10.1016/j.jpsychires.2016.10.013 27814456 PMC5191934

[B24] Guan K. Fox K. R. Prinstein M. J. (2012). Nonsuicidal selfinjury as a time-invariant predictor of adolescent suicide ideation and attempts in a diverse community sample. *J. Consult. Clin. Psychol.* 80 842–849. 10.1037/a0029429 22845782 PMC3458144

[B25] Guerreiro D. F. Cruz D. Frasquilho D. S. José C. Figueira M. L. Sampaio D. (2013). Association between deliberate self-harm and coping in adolescents: a critical review of the last 10 years\” literature. *Arch. Suicide Res.* 17 91–105. 10.1080/13811118.2013.776439 23614483

[B26] Guoliang Y. Yu W. (2018). Social transformation: a study on the structure and characteristics of mental health of junior high school students. *J. Southwest Univ. Natl. Hum. Soc. Sci. Edn.* 317 219–225.

[B27] Guvendeger D. N. Zahmacioglu O. Ciftci D. A. Kocaman G. M. Erdogan A. (2017). Association of suicide attempts and non-suicidal self-injury behaviors with substance use and family characteristics among children and adolescents seeking treatment for substance use disorder. *Substance Use Misuse* 52 604–613. 10.1080/10826084.2016.1245745 28140729

[B28] Hao Z. Lirong L. (2004). Statistical test and control method of common method deviation. *Progr. Psychol. Sci.* 12 942–942.

[B29] Hardcastle S. J. Maxwell-Smith C. Kamarova S. Lamb S. Millar L. Cohen P. A. (2018). Factors influencing non-participation in an exercise program and attitudes towards physical activity amongst cancer survivors. *Support. Care Cancer* 26 1289–1295. 10.1007/s00520-017-3952-9 29090387

[B30] Honglei G. Dandan F. Xiaoying L. Tiansheng X. (2018). The influence of negative life events on self-injury behavior of junior high school students: there is a mediating effect of regulation. *Psychol. Dev. Educ.* 34 229–238.

[B31] Jie F. Minqiang Z. (2012). Point estimation and interval estimation of intermediate effect: multiplication integral layout, nonparametric bootstrap and mcmc method. *J. Psychol.* 44 1408–1420. 10.3724/sp.j.1041.2012.01408

[B32] Klonsky E. D. Olino T. M. (2008). Identifying clinically distinct subgroups of self-injurers among young adults: a latent class analysis. *J. Consult. Clin. Psychol.* 76 22–27. 10.1037/0022-006x.76.1.22 18229979

[B33] Klonsky E. D. Oltmanns T. F. Turkheimer E. (2003). Deliberate self-harm in a nonclinical population: prevalence and psychological correlates. *Am. J. Psychiatry* 160 1501–1508. 10.1176/appi.ajp.160.8.1501 12900314 PMC4362719

[B34] Konick L. C. Gutierrez P. M. (2005). Testing a model of suicide ideation in college students. *Suicide Life-Threat. Behav.* 35 181–192. 10.1521/suli.35.2.181.62875 15843335

[B35] Martin J. Bureau J. F. Yurkowski K. Fournier T. R. Lafontaine M. F. Cloutier P. (2016). Family-based risk factors for non-suicidal self-injury: considering influences of maltreatment, adverse family-life experiences, and parent-child relational risk. *J. Adolesc.* 49 170–180. 10.1016/j.adolescence.2016.03.015 27086083

[B36] Messer J. M. Fremouw W. J. (2008). A critical review of explanatory models for self-mutilating behaviors in adolescents. *Clin. Psychol. Rev.* 28 162–178. 10.1016/j.cpr.2007.04.006 17618024

[B37] Nadorff M. R. Porter B. Rhoades H. M. Greisinger A. J. Kunik M. E. Stanley M. A. (2014). Bad dream frequency in older adults with generalized anxiety disorder: prevalence, correlates, and effect of cognitive behavioral treatment for anxiety. *Behav. Sleep Med.* 12 28–40. 10.1080/15402002.2012.755125 23470116 PMC3690155

[B38] Nock M. K. Favazza A. R. (2009). *Nonsuicidal Self-Injury: Definition and Classification.* Washington, DC: American Psychological Association.

[B39] Oliveri A. N. Ortiz E. Levin E. D. (2018). Developmental exposure to an organophosphate flame retardant alters later behavioral responses to dopamine antagonism in zebrafish larvae. *Neurotoxicol. Teratol.* 67 25–30. 10.1016/j.ntt.2018.03.002 29559250 PMC5970957

[B40] Paivio S. C. Mcculloch C. R. (2004). Alexithymia as a mediator between childhood trauma and self-injurious behaviors. *Child Abuse Negl.* 28 339–354. 10.1016/j.chiabu.2003.11.018 15066350

[B41] Paquette D. (2004). Theorizing the father- child relationship:Mechanisms and developmental outcomes. *Hum. Dev.* 47 121–143.

[B42] Qiangqin C. (2010). *A Study on the Relationship Between Attachment, Coping Style and Social Anxiety in Adolescents.* Doctoral dissertation, Hunan normal University, Changsha.

[B43] Quanquan W. Ming W. Xia L. (2017). The mechanism and influencing factors of adolescents’ self-harm behavior: based on the perspective of emotional management. *Psychol. Dev. Educ.* 32 121–130.

[B44] Runxin L. Jianmei C. (2016). A study on the relationship between stress and suicidal ideation in junior high school students. *Campus Psychol.* 14 79–81.

[B45] Shulin C. Quanquan Z. Jiannan P. Shengsheng Z. (2000). The preliminary development of coping style scale for middle school students. *Chin. J. Clin. Psychol.* 8 211–214.

[B46] Sleuwaegen E. Houben M. Claes L. Berens A. Sabbe B. (2017). The relationship between non-suicidal self-injury and alexithymia in borderline personality disorder: “actions instead of words”. *Compr. Psychiatry* 77 80–88. 10.1016/j.comppsych.2017.06.006 28646684

[B47] Sornberger M. J. Smith N. G. Toste J. R. Heath N. L. (2013). Nonsuicidal self-injury, coping strategies, and sexual orientation. *J. Clin. Psychol.* 69 571–583. 10.1002/jclp.21947 23382055

[B48] Thomassin K. Shaffer A. Madden A. Londino D. L. (2016). Specificity of childhood maltreatment and emotion deficit in nonsuicidal self-injury in an inpatient sample of youth. *Psychiatry Res.* 244 103–108. 10.1016/j.psychres.2016.07.050 27479099

[B49] Victor S. E. Glenn C. R. Klonsky E. D. (2012). Is non-suicidal self-injury an “addiction”? A comparison of craving in substance use and non-suicidal self-injury. *Psychiatry Res.* 197 73–77. 10.1016/j.psychres.2011.12.011 22401975 PMC3625678

[B50] Watson D. (2000). *Mood and Temperament.* New York, NY: Guilford Press, 158.

[B51] Watson D. Clark L. A. Tellegen A. (1988). Development and validation of brief measures of positive and negative affect:the PANASscales. *J. Pers. Soc. Psychol.* 54 1063–1070. 10.1037/0022-3514.54.6.1063 3397865

[B52] Weihua W. (2016). *The Relationship Between Parent-Child Attachment and Self Injury Behavior of Stay-at-home Children: The Role of Social Self-Efficacy and Emotional Regulation.* Changsha: Hunan Normal University.

[B53] Wendao L. Hong Z. Xia Z. (2008). Study on the identity development of college students and their relationship with parent-child attachment. *J. Capital Norm. Univ.* 1, 113–119.

[B54] Wenfeng C. Jianxin Z. (2004). The structure and validity of the Chinese version of the positive/negative emotion scale. *Chin. J. Mental Health* 18 763–765.

[B55] Xiaoyan J. Xuanwen L. Xiaoyi F. (2011). The relationship between adolescent parents, peer attachment and social adaptability. *Psychol. Dev. Educ.* 27 174–180.

[B56] Yan W. (2016). Analysis and countermeasures of the influence of parent-child attachment on children’s development. *Moderniz. Educ.* 23 173–174.

[B57] Yan Z. Tingting L. Huarong W. Lu D. Danyang L. Yangen Z. (2018). The relationship between parent-child attachment and negative emotion of college students: the multiple intermediary effects of interpersonal adaptation and mobile phone dependence. *Modern Prevent. Med.* 45 110–113.

[B58] Ying S. Fangbiao T. Wang X. (2013). Video time, mental sub-health and self-injury behavior of middle school students. *Chin. J. Mental Health* 27 468–472.

[B59] Yinqgian W. Hong Z. Ke H. Mingzhu W. Yulong T. Bin P. (2015). The relationship between parent-child attachment, peer attachment and adolescents’ negative emotion: a mediating model with regulation. *Psychol. Dev. Educ.* 32 226–235.

[B60] Yulong W. (2015). The effect of invalid family environment on self-injury behavior of different family types of adolescents. *Special Educ. China* 005 80–84.

[B61] Yulong W. Huiling C. Yalan Q. Xiuyun L. (2019). The self-punishment function of teenagers’ self-harm behavior: does it stem from guilt or shame? *Psychol. Dev. Educ.* 35 219–226.

[B62] Yulong W. Yalan Q. Liang X. Xiuyun L. (2016). The relationship between parental conflict and adolescent self-injury: a regulated intermediary model. *Psychol. Dev. Educ.* 32 377–384.

[B63] Zborowski M. J. McNamara P. (1998). Attachment hypothesis of rem sleep toward an integration of psychoanalysis, neuroscience, and evolutionary psychology and the implications for psychopathology research. *Psychoanal. Psychol.* 15 115–140. 10.1037/0736-9735.15.1.115

